# Marked seasonal variation in the wild mouse gut microbiota

**DOI:** 10.1038/ismej.2015.53

**Published:** 2015-05-29

**Authors:** Corinne F Maurice, Sarah CL Knowles, Joshua Ladau, Katherine S Pollard, Andy Fenton, Amy B Pedersen, Peter J Turnbaugh

**Affiliations:** 1 FAS Center for Systems Biology, Harvard University, Cambridge, MA, USA; 2 Centre for Immunity, Infection and Evolution (CIIE), School of Biological Sciences, University of Edinburgh, Edinburgh, UK; 3 Department of Life Sciences, Imperial College London, Silwood Park Campus, Buckhurst Road, Ascot, Berkshire, UK; 4 Gladstone Institutes, San Francisco, CA, USA; 5 Institute for Human Genetics and Division of Biostatistics, University of California San Francisco, San Francisco, CA, USA; 6 Institute of Integrative Biology, University of Liverpool, Biosciences Building, Liverpool, UK; 7 Department of Microbiology and Immunology, G.W. Hooper Research Foundation, University of California San Francisco, San Francisco, CA, USA

## Abstract

Recent studies have provided an unprecedented view of the microbial communities colonizing captive mice; yet the host and environmental factors that shape the rodent gut microbiota in their natural habitat remain largely unexplored. Here, we present results from a 2-year 16 S ribosomal RNA gene sequencing-based survey of wild wood mice (*Apodemus sylvaticus*) in two nearby woodlands. Similar to other mammals, wild mice were colonized by 10 bacterial phyla and dominated by the Firmicutes, Bacteroidetes and Proteobacteria. Within the Firmicutes, the *Lactobacillus* genus was most abundant. Putative bacterial pathogens were widespread and often abundant members of the wild mouse gut microbiota. Among a suite of extrinsic (environmental) and intrinsic (host-related) factors examined, seasonal changes dominated in driving qualitative and quantitative differences in the gut microbiota. In both years examined, we observed a strong seasonal shift in gut microbial community structure, potentially due to the transition from an insect- to a seed-based diet. This involved decreased levels of *Lactobacillus*, and increased levels of *Alistipes* (Bacteroidetes phylum) and *Helicobacter*. We also detected more subtle but statistically significant associations between the gut microbiota and biogeography, sex, reproductive status and co-colonization with enteric nematodes. These results suggest that environmental factors have a major role in shaping temporal variations in microbial community structure within natural populations.

## Introduction

Mammals are home to trillions of microbes in their gastrointestinal tract (the gut microbiota), which impact multiple aspects of host health and disease (Sommer and Backhed, 2013). Elucidating the ecological and evolutionary processes that shape host-associated microbial communities remains a major outstanding goal (Costello *et al.,* 2012). Laboratory rodents are a valuable tool to dissect the relative contributions of intrinsic and extrinsic factors (Carmody *et al.,* 2015); however, it remains unclear whether these interactions can be generalized to mammals in their natural habitat. Recent studies have provided an initial view into the ecological factors linked to inter-individual variations in the gut microbiotas of wild animals. Comparative analyses suggest that diet is a major environmental factor contributing to gut microbial variation between mammalian species (Muegge *et al.,* 2011). Diet also shapes the gut microbiota within a species, as evidenced by longitudinal analyses of the black howler monkey gut microbiota (Amato *et al.,* 2013, 2015) and dietary perturbation experiments in wild-caught mice and fish (Bolnick *et al.,* 2014; Wang *et al.,* 2014). Biogeographic variation in the gut microbiota at large spatial scales has also been reported in house mice (Linnenbrink *et al.,* 2013). Finally, host-specific factors like co-colonization with enteric parasites (Hayes *et al.,* 2010; Keeney and Finlay, 2011) and host genetics (Benson *et al.,* 2010; Ochman *et al.,* 2010; McKnite *et al.,* 2012; Goodrich *et al.,* 2014) may also contribute to inter-individual and temporal variations in gut microbial community structure.

Yet, the relative strengths of these various factors, and their interactions, remain unclear owing to the lack of systematic analyses that monitor both intrinsic and extrinsic factors in natural populations. Such an analysis would require tractable systems wherein host factors, environmental parameters and temporal variations in the gut microbiota can be monitored *in situ*. Here, we report findings from such a study in well-characterized populations of wood mice (*Apodemus sylvaticus*) in the United Kingdom, which we monitored for 2 years. We simultaneously measured multiple environmental (season, location, population density) and host (age, sex, reproductive status, parasite infection status) parameters, and repeatedly sampled multiple individuals over time. Using these data, we examine the relative importance of environmental and intrinsic host factors in shaping gut microbial community variation between and within-individuals over time. We discovered a notable seasonal variation in gut microbial community structure, which we propose is caused by changes in host dietary intake. We also found evidence for an impact of spatial structure over a smaller scale than previously reported, reproductive status and nematode colonization. Together, our results provide an initial view of the wild wood mouse gut microbiota and support the hypothesis that environmental factors such as changes in food availability and subsequent dietary intake have a dominant role in shaping wild mammal gut microbial communities.

## Materials and methods

### Sample collection

In 2010 and 2011, *A. sylvaticus* were trapped on six grids in two mixed woodlands (Manor and Haddon Wood; Supplementary Figure S1) on the Wirral peninsula, UK. On each grid, two live traps baited with grain and bedding material were placed every 10 meters in a 70 m × 70 m square, and trapped monthly from May to November for three consecutive nights in both years. In 2011, trapping was also performed for two consecutive nights during one additional week in each of the months August, September, October and November, though no treatments were given. Trapped animals were tagged using subcutaneous passive integrated transponder tags, so they could be individually identified upon recapture. Fecal samples were collected from all traps containing a single animal and stored in 10% buffered formalin for identification of gut parasites (Knowles *et al.,* 2013). A sub-sample was also collected for characterization of the gut microbiota, which was frozen at −80^o^C within 8 h of collection. To assess the potential effect of overnight temperature on gut microbial communities, we retrieved temperature data for each sampling night from the Hawarden Chester airport weather station near our field sites between the hours of 6pm and 12pm the following day, the time from which mice could enter traps to when we collected fecal samples.

### Host phenotyping

Animals were aged as either juvenile, sub-adult or adult according to pelage in the first instance, with body mass used as a secondary trait where pelage was inconclusive (juvenile <12 g, sub-adult: 12–16 g, adult >16 g). Body length, weight, sex and reproductive status were recorded. Animals were characterized as being either reproductively active (descended or protruding testes for males, pregnant or with a perforate vagina for females) or inactive. A subset of the mice was given antiparasitic treatments, including Ivermectin and Toltrazuril (2010), and Ivermectin, Fipronil, Pyrantel pamoate, or two-drug combinations (2011). We did not detect any significant impact of treatment on the gut microbiota (Table 1). Blood samples were tested for *Bartonella* using a nested PCR assay (Knowles *et al.,* 2013).

### 16 S rRNA gene sequencing and analysis

16 S ribosomal RNA (rRNA) gene sequencing was performed on fecal samples collected from each trap to characterize the distal gut microbiota (*n*=481 samples, 196 555±24 236 sequences per sample; Supplementary Table S1). DNA was extracted using the PowerSoil bacterial DNA extraction kit (MoBio, Carlsbad, CA, USA), and the V4 region of the 16 S rRNA gene was PCR-amplified in triplicate using custom barcoded universal bacterial primers with the following protocol: 94 °C for 3 min, 35 cycles of 94°C for 45 s, 50 °C for 30 s, and 72 °C for 90 s, with a final extension at 72 °C for 10 min (Maurice *et al.,* 2013). Triplicates were pooled, confirmed by gel electrophoresis, cleaned with the Ampure XP kit (Agencourt, Danvers, MA, USA), quantified using the Quant-iT Picogreen dsDNA Assay Kit (Invitrogen, Carlsbad, CA, USA), and sequenced on the Illumina HiSeq platform. 16 S rRNA gene sequences were analyzed using the QIIME software package (Caporaso *et al.,* 2010). All sequences were used for the comparison of the relative abundance of bacterial taxa. Operational taxonomic units were assigned at 97% similarity against the Greengenes database (DeSantis *et al.,* 2006), which we trimmed to span only the 16 S rRNA region flanked by our sequencing primers (positions 521–773). LefSe (Segata *et al.,* 2012a) was run on sub-sampled data sets, after filtering out species-level phylotypes with <100 sequences or found in only one sample. Statistical analysis of Bray–Curtis dissimilarities calculated using the relative abundance of bacterial genera was conducted using RStudio (ver. 0.98.1091) and the adonis function in the R package ‘vegan' (Oksanen *et al.,* 2015). Only the first sample was included for each mouse to avoid artifacts caused by within animal comparisons. Significance values were computed using 10 000 permutations.

### Parasite diagnosis

Gastrointestinal parasites (nematodes, cestodes and *Eimeria* protozoa) were detected using the salt flotation technique (Pritchard and Kruse, 1982). Saturated salt solution was added to formalin-preserved fecal samples, such that eggs and oocysts in each sample could be concentrated on a coverslip, and scanned for parasite detection at × 10 magnification. × 40 magnification was used for parasite identification. Coccidia (parasites belonging to the genus *Eimeria*) were identified using unsporulated oocyst morphology (Nowell and Higgs, 1989), and helminths using egg morphology. For each parasite species, the number of eggs or oocysts per gram of feces was calculated for each sample. When multiple samples were present for an individual within a 3-day trapping period, the arithmetic mean egg/oocyst count was taken across these days. The dominant parasites detected were nematodes (largely *Heligmosomoides polygyrus*) and coccidia, and thus our analyses focus on these two parasite groups.

### Linear mixed models

We performed linear mixed models using the lme4 package in R v.3.0.1 (Bates *et al.,* 2014). We controlled for repeated sampling of individual mice by including individual ID as a random intercept term. Model assumptions were checked by examining the distribution of residuals and plotting fitted values against residuals; response variables were square root or log-transformed where necessary to ensure model assumptions were met. For models of individual genera, only samples with non-zero abundance were included. In all starting models, the same set of predictors was included: external overnight temperature, grid, month, year, age, sex, nematode infection status, *Eimeria* infection status, drug treatment and reproductive status. Several interaction terms were included: year by month; reproductive status by sex; and parasite infection variables by treatment. Only samples for which full metadata on all the above metrics were available were included (Supplementary Table S2). All models were initially simplified by backwards-stepwise elimination of terms with *P*-value >0.10, beginning with interactions, and the final minimal model included only terms with *P*-value <0.05. Adjusted *P-*values (*q*-values) were calculated based on the 'Graphically Sharpened' false discovery rate method (Pike, 2011).

### Spatial structuring of microbial communities

As wood mice are territorial and have home ranges smaller than our trapping grids (Godsall *et al.,* 2014), fine-scale spatial variation in microhabitat and food availability could influence gut microbial ecology, both within and across our trapping grids. To test for biogeographic effects at this scale, we examined spatial autocorrelation in the gut microbiota according to mouse capture location. Spatial autocorrelations were measured using the Moran's I statistic (Moran, 1950). Only the first sample was included for each mouse to avoid artifacts caused by within animal comparisons. Genera found in ⩾10 samples (or mice) were analyzed, along with the first principal coordinates from our Bray–Curtis, unweighted UniFrac and weighted UniFrac analyses. We used a binary spatial weights matrix, with spatial neighborhoods defined as being 0–50 m apart. Data from Manor and Haddon woods were analyzed both together and separately. To control for temporal trends, we restricted our analysis to samples collected between August and November and analyzed the 2 years separately. Spatial weight matrices were row-standardized. The significance of Moran's *I* values was assessed with permutation tests, coded using a Markov chain Monte Carlo algorithm. For each *P*-value, 10 chains of length 1 000 000 were run, each starting from a random initial permutation. These settings were judged to give good chain convergence based on examination of running mean plots. We used the software packages GeoDa and PySAL (https://geodacenter.asu.edu). Batch scripts/code are available on request.

## Results

### The wild mouse gut harbors abundant Lactobacilli and putative enteric pathogens

**Figure 1:**
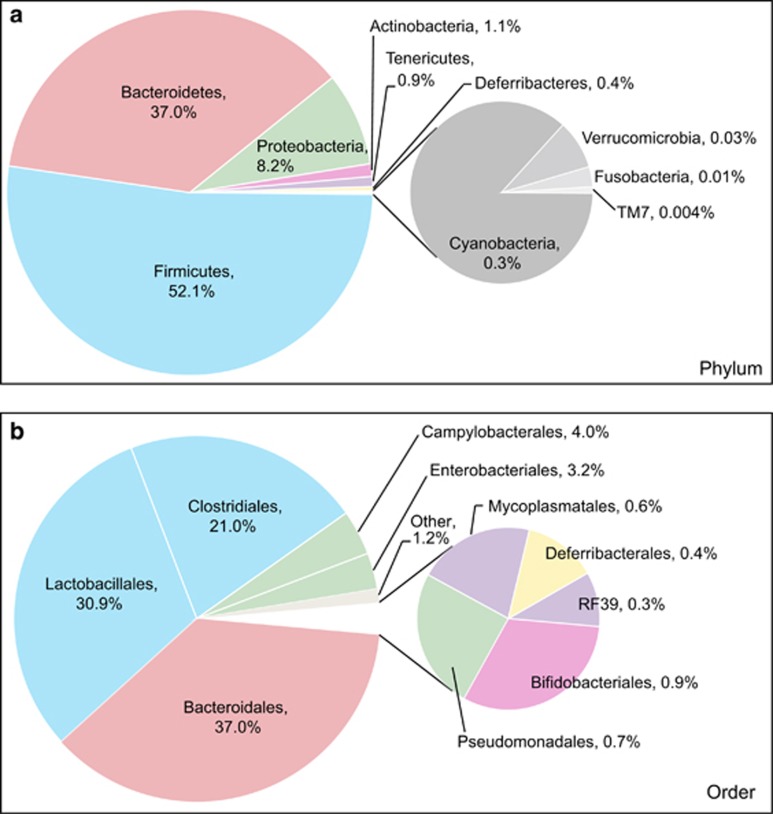
Taxonomic analysis of the wild mouse gut microbiota. Pie charts represent the relative abundance of bacterial (**a**) phyla and (**b**) orders (*n*=481 samples). The 10 most abundant phyla and orders are shown (phyla with a mean abundance <0.001% are not included; the remaining orders are represented by the ‘other' slice). Taxa are colored based on phylum. Sequences within the Cyanobacteria phylum could be attributed to chloroplasts (order Streptophyta), non-photosynthetic bacteria related to Cyanobacteria that are common in the mammalian gut (order YS2) (Di Rienzi *et al.,* 2013), and algae (order Chlorophyta, family Trebouxiophyceae). We did not detect any consistent seasonal changes in the abundance or prevalence of these three groups.

Consistent with results in captive and wild mammals (Ley *et al.,* 2008a), wild wood mice were colonized by 10 bacterial phyla: Firmicutes (52.1±1.0% 16 S rRNA gene sequences; mean±s.d.), Bacteroidetes (37.0±0.9%), Proteobacteria (8.2±0.5%), Actinobacteria (1.1±0.2%), Tenericutes (0.9±0.1%), Deferribacteres (0.4±0.1%), Cyanobacteria (0.3±0.03%), Verrucomicrobia (0.03±0.03%), Fusobacteria (0.01±0.01%) and TM7 (0.004±0.0004%) (Figure 1a). Within the Firmicutes, the dominant bacterial order was the Lactobacillales (genus: *Lactobacillus*) (Figure 1b, Supplementary Figure S2). We also observed multiple α-, ɛ- and γ-proteobacterial genera that include potential bacterial pathogens: for example, *Bartonella*, *Helicobacter, Pseudomonas, Rickettsiella* and *Yersinia* (Figure 1b, Supplementary Figure S2, Supplementary Table S3). All nine of the mice with detectable fecal *Bartonella* also tested positive in time-matched blood samples, leading to a significant association between blood and fecal detection of this genus (*P*-value<0.05, *χ*^2^-test). Many of these genera were widespread, most notably *Helicobacter* (97.9% of samples), *Pseudomonas* (73.2%) and *Yersinia* (44.5%). The same was true for intestinal parasites (Supplementary Table S4), including *H. polygyrus* (40%) and *Eimeria hungaryensis* (29.3%).

### Marked seasonal variation in microbial community structure

**Figure 2:**
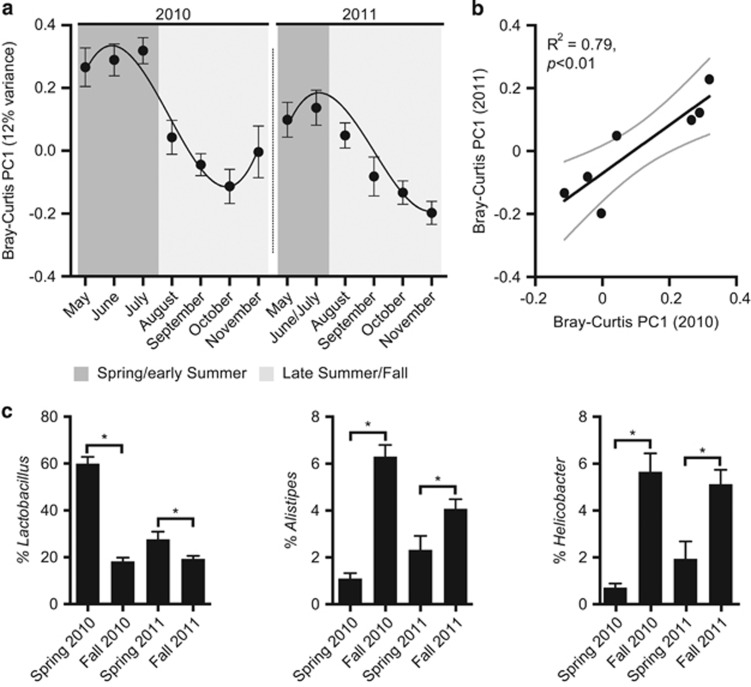
Seasonal variations in the wild mouse gut microbiota. (**a**) The first principle coordinate from a Bray–Curtis-based analysis of microbial community structure over time. Trend lines were generated by fitting a polynomial function to values from each year (GraphPad Prism version 6.0). Values are mean±s.e.m. (*n*=14–80 samples per group). Values from June and July were combined in 2011 owing to limited available samples in July (*n*=2). (**b**) Association between average monthly microbial community structures between years. Values are mean (thick black line) and 95% CI (thin grey lines) from a linear regression. (**c**) The relative abundance of bacterial genera in spring and fall of both years. Values are mean±s.e.m. (*n*=24–123 samples per group; the first sample from each mouse was included). Asterisks represent significant differences (*P*-value<0.05, Wilcoxon rank-sum test).

Analysis of Bray–Curtis dissimilarity among samples revealed a clear seasonal pattern differentiating samples collected in the spring/early summer (May through July) and those collected in late summer/fall (August through November) (Figure 2a, Supplementary Figure S3; *P*-value<0.001, PERMANOVA of Bray–Curtis distances). The observed seasonal shift in the microbiota coincides with the expected timing of an annual transition to a seed-based diet from a more insect-based diet (Watts, 1968), and may therefore be driven by a seasonal shift in food availability and diet. Consistent with this hypothesis, the mean microbial community structure for each month was significantly correlated between the 2 years (Figure 2b; *R*^2^=79%, *P*-value<0.01). The association between season and microbial community structure was significant in both years when considered independently, although the difference was more marked in 2010 (pseudo *F*-value=31.2 (2010) versus 9.9 (2011), *P*-value<0.001 for both years; PERMANOVA test). Statistical analysis with the LefSe software package revealed taxonomic groups ranging from the phylum to genus level that were consistently associated with season in both years (Supplementary Table S5). *Lactobacillus* was found at a significantly higher abundance in the spring of both years, whereas *Alistipes* and *Helicobacter* were consistently enriched in the fall (Figure 2c).

**Figure 3:**
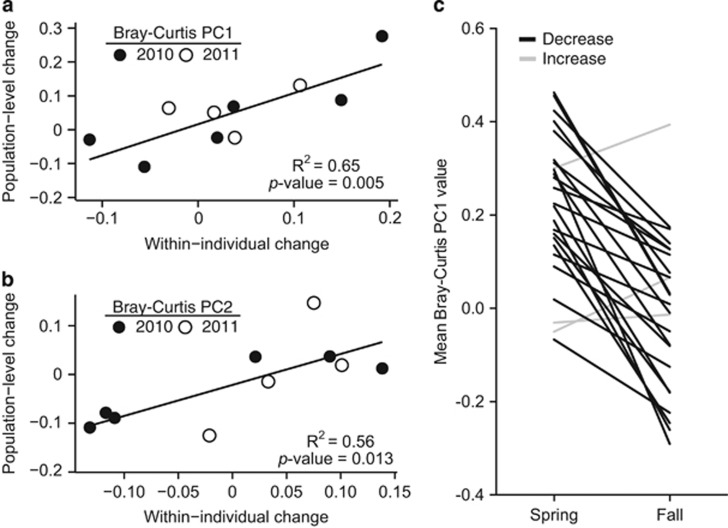
Seasonal patterns are detectable within-individuals captured multiple times. Correlations between the mean month-to-month change in Bray–Curtis principle coordinates 1 (panel **a**) and 2 (panel **b**) within-individuals, relative to the monthly change observed at the population level (including only one pair of observations per mouse; *n*=2–11 paired samples per datapoint). Dots represent monthly changes seen in 2010 (black) and 2011 (white). See Supplementary Figure S4 for plots of individual animals over time. (**c**) We calculated the mean value of Bray–Curtis principal coordinate 1 value for each mouse in Season 1 (spring/early summer) and 2 (late summer/fall) (*n*=25 mice; less than equal to one sample per mouse per month included). Nearly all mice exhibited a consistent direction of change (black lines), with the exception of three animals (grey lines).

Analysis of mice captured multiple times within a year confirmed that these microbial changes occurred within individuals and were not simply due to mouse population turnover (that is, seasonal changes in the types of individual captured). We observed within individual shifts in microbiota structure in both years of the study that followed the overall population trend (Supplementary Figure S4). This was reflected by a strong positive correlation between month-to-month differences in the mean population-wide value for Bray–Curtis principal coordinates 1 and 2 (excluding repeat captured individuals) and the mean within-individual change in these metrics (PC1 *R*^2^=65% PC2 *R*^2^=56% both *P*-value<0.05, linear regression; Figures 3a and b). Analysis of 25 mice captured in both seasons confirmed that in nearly all cases there was a consistent direction of change (Figure 3c; *P*-value<0.0001, Wilcoxon rank-sum test).

### Limited spatial heterogeneity in community structure

Although microbial community structure differed significantly between the two woodlands (*P*-value<0.001, PERMANOVA of Bray–Curtis dissimilarities), this effect was noticeably weaker than that of season: pseudo *F*-value=35.3 (season) versus 4.6 (wood) when considering both years. Consistent with this weak effect, LefSe analysis only identified two nested taxa that were significantly enriched in Manor Wood: the Clostridia class and the Clostridiales order (LDA>2, *P*-value<0.05). We did not detect any taxa that were significantly enriched in Haddon Wood.

**Figure 4:**
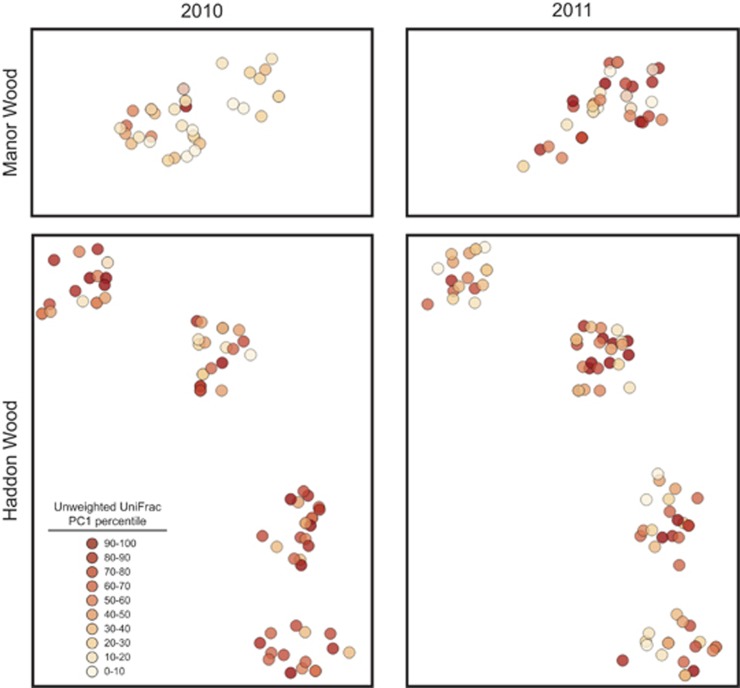
Spatial distribution of microbial community structure. Each circle represents the physical location of a given mouse at the time of sampling in Manor or Haddon Wood, which are subdivided into two and four trapping grids, respectively. Shading is proportional to the percentile along unweighted UniFrac principal coordinate 1 (an indication of overall microbial community membership). Between August and November in 2010 there was a slight but significant difference in community composition between Haddon and Manor woods. However, this difference was absent in August to November 2011. Within woods, no significant spatial structuring of communities was observed in either year.

To quantify the spatial structure of the wild mouse gut microbiota in more detail, we evaluated spatial autocorrelation at the genus level and using community dissimilarity metrics (see Methods). In 2010, we detected significant spatial autocorrelation for the Bray–Curtis and unweighted UniFrac metrics (Figure 4; *q*-value<0.01). However, these patterns were weaker and only present for unweighted UniFrac in 2011, and were absent in all cases when we only considered samples from Haddon or Manor wood. Similarly, analyses of bacterial genera failed to detect significant spatial autocorrelation for 117 of the 117 tested groups during either year (*q*-value<0.01). Moreover, the maximum Moran's *I* value for this distance class was 0.138, further indicating nonexistent or weak spatial associations. Together, these analyses suggest that although the overall pattern of microbial community structure was distinct between Haddon and Manor Wood, there was no evidence for finer spatial structure within woods or between individual bacterial genera.

### Multivariate modeling reveals associations with both host and environmental factors

We next used linear mixed models (see Methods) to tease apart the relative influence of multiple environmental and host factors, and to determine their effects in isolation of confounding factors. We constructed six models for community dissimilarity metrics (principal coordinates 1 and 2 for Bray–Curtis, unweighted UniFrac and weighted UniFrac) as well as separate models for the 10 most abundant bacterial genera (Table 1). Overall, these analyses suggest that the wood mouse gut microbiota is primarily shaped by environmental factors, with significant evidence for both temporal (see ‘Year' and ‘Month' columns) and spatial structuring (see ‘Grid' column). These temporal trends could not simply be explained by seasonal variation in temperature (Supplementary Figure S5), as they were unaltered by inclusion of overnight temperature as a covariate (Table 1). For all community dissimilarity metrics examined and most individual genera, our minimal models included a significant year by month interaction term, indicating seasonal differences that varied somewhat across the 2 years investigated. If these interaction terms were dissolved into their component terms, strong main effects of month were observed in nearly all models, with effects of year also common though generally weaker. Consistent with our prior analysis of spatial autocorrelation, there was a strong association between the community dissimilarity metrics and trapping grid with weaker associations at the genus level. We also detected associations between some metrics and local population density at the time of capture (Table 1).

**Figure 5:**
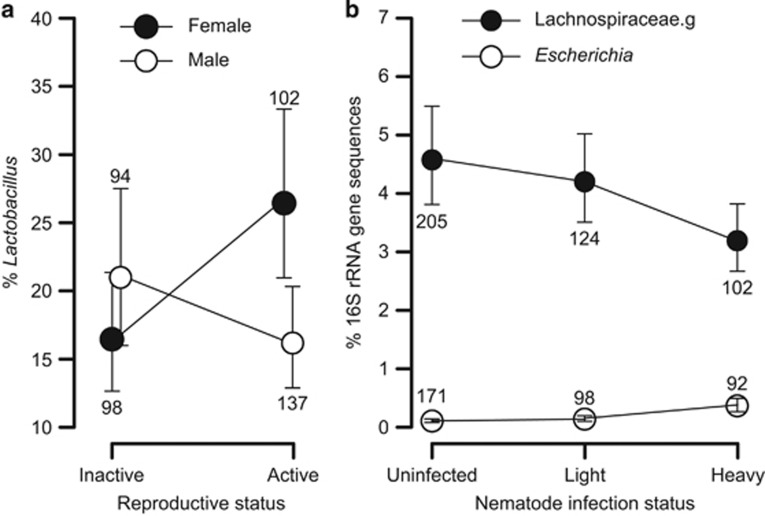
The gut microbiota is associated with intestinal helminth infection and reproductive state. (**a**) Values represent the relative abundance of *Lactobacillus* according to host sex and reproductive status. (**b**) Nematode infection is positively associated with *Escherichia* and negatively associated with an unclassified genus within the Lachnospiraceae family. All samples with non-zero abundance were included. Values are mean±s.e.m. (*n*=92–205 samples per group).

To a lesser extent than extrinsic factors like season and year, host factors such as reproductive status and sex were associated with microbial community structure, sometimes in the form of an interaction between these two terms (Table 1). For example, the abundance of *Lactobacillus* was higher in reproductively active than non-active females, but did not depend on reproductive status for males (Figure 5a). We also detected associations between the gut microbiota and intestinal parasites. In particular, nematode infections were inversely associated with the abundance of the most abundant *Lachnospiraceae* genus and positively associated with the genus *Escherichia* (Figure 5b). However, no significant associations between coccidia infection or anti-parasite treatment and the gut microbiota were found, possibly due to the transient nature of the intervention (monthly treatment intervals; see Methods). Age-related differences were rare, with *Alistipes* the only 1 of the 10 most abundant bacterial genera associated with host age, showing an increase across the age groups from juvenile to adult (Table 1).

To illustrate how much variation in Bray–Curtis principal coordinates 1 and 2 was explained by environmental factors like month and year, compared with host-related factors, we calculated marginal R^2^ statistics from our linear mixed models (Nakagawa and Schielzeth, 2013). These are equivalent to classic R^2^ statistics for linear models, indicating the percentage of variation explained by a given set of predictor variables (fixed effects). For both Bray–Curtis PC1 and PC2, month (that is, seasonal differences) explained a much larger proportion of variance than year (Supplementary Table S6). Inclusion of year when month was already present in the model provided little additional explanatory power (PC1: R^2^_GLMM(m)_ =43.9% with month only versus 48.7% with month and year; PC2: R^2^_GLMM(m)_=10.1% with month only versus 10.5% with month and year). Furthermore, allowing the seasonal effect to vary among years (by inclusion of a month × year interaction term) yielded limited additional explanatory power for PC1 (R^2^_GLMM(m)_ =52% versus 49% variance explained), with a twofold increase in variance explained for PC2 (R^2^_GLMM(m)_ =19.5% versus 10% variance explained).

Thus, seasonal differences in the gut microbiota appear to dominate the differences between years and are largely consistent across years, in agreement with our earlier analyses (Figure 2). Host-related factors (age, sex, reproductive status), enteric parasite infections and host density explained some additional variance (12% more for PC1 and 8% more for PC2 than models with only month and year terms), though their contribution was again smaller than the strong seasonal effects, particularly for PC1 (Supplementary Table S6). Individual identity explained 18% of the variation in Bray–Curtis PC1 even after including all other factors. We confirmed these trends by analyzing the entire Bray–Curtis dissimilarity matrix according to season, host sex, and wood (see Methods). Although all three factors showed a significant effect, seasonal effects explained more variation (*R*^2^=13.3%, *P*-value<10^−4^) than either host sex (*R*^2^=0.8%, *P*-value<0.05) or spatial structure (*R*^2^=0.8%, *P*-value<0.05).

## Discussion

At the phylum level, the wild mouse gut microbiota is comparable to that of other mammals (including humans) with two major groups, the Firmicutes and Bacteroidetes, accounting for ~90% of the 16 S rRNA gene sequencing reads (Ley *et al.,* 2008a; Muegge *et al.,* 2011). We also detected high levels of the *Lactobacillus* genus (phylum: Firmicutes; order: Lactobacillales) constituting up to one-third of the community, similar to other omnivorous mammals, such as bears, squirrels and lemurs (Supplementary Figure S6). These results confirm that to a large degree the mammalian gut microbiota assembles in a reproducible fashion regardless of the host species (Ley *et al.,* 2008a; Muegge *et al.,* 2011), reflective of the restricted set of microorganisms that have adapted to life in the gastrointestinal tract (Ley *et al.,* 2008b).

In contrast to ‘specific pathogen free' laboratory mice, we detected widespread colonization by bacterial taxa that contain enteric pathogens, including *Helicobacter* and other Proteobacteria. However, given the resolution of our sequencing methods and the limited studies of wild mouse pathogens we cannot exclude the fact that these are commensal strains. Despite this important caveat, our results are consistent with previous reports, indicating that wild house mice can be reservoirs of diverse *Helicobacter* strains capable of infecting humans and other vertebrates (O'Rourke *et al.,* 2001; Parker *et al.,* 2009; Wasimuddin *et al.,* 2012). We observed that *Helicobacter* abundance increased in late summer/fall, when *Lactobacillus* levels are low. This might suggest that *Lactobacillus* confers protection against infection as has been demonstrated in laboratory mice (Kabir *et al.,* 1997; Pena *et al.,* 2005; Medellin-Pena and Griffiths, 2009; Eaton *et al.,* 2011). Alternatively, immune status (that is, IL-22 deficiency) has been linked to the abundance of *Lactobacillus* (Zenewicz *et al.,* 2013), potentially suggesting that these seasonal changes might be in part driven by the host response to bacterial infection. Additional studies will be necessary to determine how the immune system of these mice tolerates long-term enteric pathogen colonization and to characterize the reciprocal interactions between these enteric pathogens and the commensal gut microbiota.

The wild mouse gut microbiota underwent a consistent seasonal shift in both years, with a decrease in *Lactobacillus* and concomitant increases in *Alistipes*, *Helicobacter* and the Lachnospiraceae family (phylum: Firmicutes). A possible explanation is that mid-summer represents a transition from a diet rich in insects to a diet primarily composed of seeds (Watts, 1968), coincident with the annual seed fall, which usually starts in late July in UK woodlands (Gurnell, 1993). Thus, we propose that seasonal patterns in dietary intake drive variations in the gut microbial community structure of wild wood mice. Differences in the timing, extent and tree species composition of seed fall, which can vary markedly between years (Gurnell, 1993), may explain the observed variation between years in the magnitude of the seasonal microbiota transition observed. Notably, a recent study of rural human subjects from South Dakota revealed differences in the gut microbiota in summer relative to winter (Davenport *et al.,* 2014), suggesting that seasonal reconfigurations may be a conserved feature of host-associated microbial communities.

If diet is indeed the dominant factor it still remains unclear what specific components of the diet might drive the observed changes to gut microbial community structure. The elevated levels of *Alistipes* in the fall may be reflective of increased bile acid levels triggered by an increased consumption of fat, as seen in a recent human dietary intervention study (David *et al.,* 2014). Members of the Lachnospiraceae family, including *Eubacterium rectale* and *Roseburia*, have been linked to the fermentation of dietary plant polysaccharides in human studies (David *et al.,* 2014; Duncan *et al.,* 2007), and were also enriched in the fall coinciding with the increased access to plant seeds. Similarly, the source and/or dietary trigger of *Lactobacillus* (often a minor member of the mammalian distal gut microbiota) also remains unclear. *Lactobacillus* is often found in fermented foods (Wolfe *et al.,* 2014), raising the possibility that their elevated abundance early in the year may be driven by its cultivation in wood mouse food stores over winter.

Alternatively, seasonal changes in mouse physiology, including torpor and reduced food consumption during winter, could also have a role in the observed seasonal trends. Indeed, seasonal restructuring of the gut microbiota has recently been observed in ground squirrels under controlled laboratory conditions (Carey *et al.,* 2013). These shifts coincided with hibernation, suggesting they are driven by a shift from dietary to host-derived substrates. We detected similar patterns in wild rodents, including a decrease in the relative abundance of *Lactobacillus* and an increase in *Alistipes* from spring/early summer to late summer/fall. Although wood mice do not hibernate, they are subject to daily torpor in conditions of low temperature and food restriction. Thus, it is possible that the seasonal microbial shifts seen here may be driven by the transition to a state of intermittent torpor.

What are the potential consequences of the observed seasonal shifts in gut microbial community structure? Recent human intervention studies have shown rapid and reproducible changes in microbial community structure and function upon consumption of an animal- versus plant-based diet (David *et al.,* 2014). These results, considered together with the current findings from wild wood mice, make it tempting to speculate that the mammalian gut microbiota may provide a rapid way to optimize caloric intake given volatile shifts in the availability of different foods. Microbial communities that could rapidly shift their metabolic activity in response to changes in host dietary intake could have enhanced dietary flexibility, likely increasing the fitness of the host and its microbial consortia.

We also found significant but weak evidence for spatial structure, unlike the more robust associations with geographic region found in recent studies of house mice (Linnenbrink *et al.,* 2013), wild primates (Degnan *et al.,* 2012) and humans (Yatsunenko *et al.,* 2012). The significant spatial structure that we did find was evident only in community-wide metrics when comparing between woods. Individual bacterial genera showed no spatial structure, and no spatial structuring was evident within woods at either the community or individual genus levels. These results emphasize that the gut microbiota of these wild mouse populations is primarily shaped by factors that are not spatially structured at the scales that we considered. These results suggest that either (i) microbial dispersal occurs efficiently over distances far greater than the host range evaluated here and/or (ii) the observed bacterial taxa are long-term and stable residents of the wild wood mouse gut microbiota. Strain-level analyses of the gut microbiota (Segata *et al.,* 2012b; Faith *et al.,* 2013) could help determine whether there are finer differences between woods, or among areas within each wood. Furthermore, surveying wild mice across more distant sites could provide additional insight into broader biogeographical patterns.

Our linear mixed models revealed significant associations with reproductive status and intestinal parasites. Consistent with these findings, recent studies indicate that the human gut microbiota is altered during pregnancy (Koren *et al.,* 2012), and studies in laboratory mice have shown that infection by the nematode *Trichuris muris* depends on the gut microbiota (Hayes *et al.,* 2010). The associations between intestinal nematodes and the bacterial genera *Escherichia* (positive) and *Lachnospiraceae* (negative) support recent studies in humans and animal models (Walk *et al.,* 2010; Rausch *et al.,* 2013), though we did not find the specific association between *H. polygyrus* and relative *Lactobacillus* abundance, as recently reported in laboratory mice (Reynolds *et al.,* 2014). Whether the associations found result from an altered immune response of the host or from direct interactions between the intestinal parasites and the gut microbiota remains to be elucidated. Determining the causal direction and underlying mechanisms of these interactions will require more extensive longitudinal analyses of wild mice before and after helminthic infection, as well as controlled studies using captured and/or captive mice.

In conclusion, despite the common use of laboratory mice to study the environmental and host factors that shape host-associated microbial communities, we still know very little about their natural state. Our results provide an initial view of the wild-wood mouse gut microbiota, emphasizing not only commonalities between mammals, but also the importance of considering temporal variations in nutritional status, enteric pathogens, reproductive status and parasite burden in setting the stage for host-microbial interactions. Follow-up observational and interventional studies of wild mice, paired with an in-depth analysis of dietary intake, are necessary to test the hypothesis that the observed seasonal trends are due to changes in diet, and could provide a complementary and tractable approach towards better understanding the causes and consequences of inter-individual variations in the mammalian gut microbiota.

## Figures and Tables

**Table 1:**
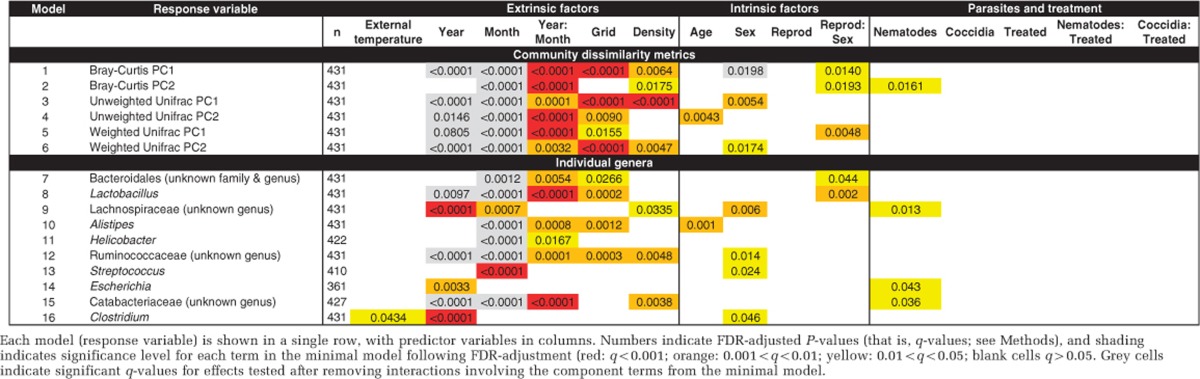
Environmental and host factors associated with microbial community structure and membership in linear mixed models

## References

[bib1] Amato KR, Yeoman CJ, Kent A, Righini N, Carbonero F, Estrada A et al. (2013). Habitat degradation impacts black howler monkey (Alouatta pigra) gastrointestinal microbiomes. ISME J 7: 1344–1353. 23486247 10.1038/ismej.2013.16 PMC3695285

[bib2] Amato KR, Leigh SR, Kent A, Mackie RI, Yeoman CJ, Stumpf RM et al. (2015). The gut microbiota appears to compensate for seasonal diet variation in the wild black howler monkey (Alouatta pigra). Microb Ecol 69: 434–443. 25524570 10.1007/s00248-014-0554-7

[bib3] Bates D, Maechler M, Bolker B, Walker S. (2014). lme4: Linear mixed-effects models using Eigen and S4. R package version 0.999999-2. http://CRAN.R-project.org/package=lme4.

[bib4] Benson AK, Kelly SA, Legge R, Ma F, Low SJ, Kim J et al. (2010). Individuality in gut microbiota composition is a complex polygenic trait shaped by multiple environmental and host genetic factors. Proc Natl Acad Sci USA 107: 18933–18938. 20937875 10.1073/pnas.1007028107 PMC2973891

[bib5] Bolnick DI, Snowberg LK, Hirsch PE, Lauber CL, Knight R, Caporaso JG et al. (2014). Individuals' diet diversity influences gut microbial diversity in two freshwater fish (threespine stickleback and Eurasian perch). Ecol Lett 17: 979–987. 24847735 10.1111/ele.12301 PMC4084827

[bib6] Caporaso JG, Kuczynski J, Stombaugh J, Bittinger K, Bushman FD, Costello EK et al. (2010). QIIME allows analysis of high-throughput community sequencing data. Nat Methods 7: 335–336. 20383131 10.1038/nmeth.f.303 PMC3156573

[bib7] Carey HV, Walters WA, Knight R. (2013). Seasonal restructuring of the ground squirrel gut microbiota over the annual hibernation cycle. Am J Physiol Regul Integr Comp Physiol 304: R33–R42. 23152108 10.1152/ajpregu.00387.2012 PMC3543654

[bib8] Carmody RN, Gerber GK, Luevano Jr JM, Gatti DM, Somes L, Svenson KL et al. (2015). Diet dominates host genotype in shaping the murine gut microbiota. Cell Host Microbe 17: 72–84. 25532804 10.1016/j.chom.2014.11.010 PMC4297240

[bib9] Costello EK, Stagaman K, Dethlefsen L, Bohannan BJ, Relman DA. (2012). The application of ecological theory toward an understanding of the human microbiome. Science 336: 1255–1262. 22674335 10.1126/science.1224203 PMC4208626

[bib10] Davenport ER, Mizrahi-Man O, Michelini K, Barreiro LB, Ober C, Gilad Y. (2014). Seasonal variation in human gut microbiome composition. PLoS One 9: e90731. 24618913 10.1371/journal.pone.0090731 PMC3949691

[bib11] David LA, Maurice CF, Carmody RN, Gootenberg DB, Button JE, Wolfe BE et al. (2014). Diet rapidly and reproducibly alters the human gut microbiome. Nature 505: 559–563. 24336217 10.1038/nature12820 PMC3957428

[bib12] Degnan PH, Pusey AE, Lonsdorf EV, Goodall J, Wroblewski EE, Wilson ML et al. (2012). Factors associated with the diversification of the gut microbial communities within chimpanzees from Gombe National Park. Proc Natl Acad Sci USA 109: 13034–13039. 22826227 10.1073/pnas.1110994109 PMC3420156

[bib13] DeSantis TZ, Hugenholtz P, Larsen N, Rojas M, Brodie EL, Keller K et al. (2006). Greengenes, a chimera-checked 16 S rRNA gene database and workbench compatible with ARB. Appl Environ Microbiol 72: 5069–5072. 16820507 10.1128/AEM.03006-05 PMC1489311

[bib14] Di Rienzi SC, Sharon I, Wrighton KC, Koren O, Hug LA, Thomas BC et al. (2013). The human gut and groundwater harbor non-photosynthetic bacteria belonging to a new candidate phylum sibling to Cyanobacteria. Elife 2: e01102. 24137540 10.7554/eLife.01102 PMC3787301

[bib15] Duncan SH, Belenguer A, Holtrop G, Johnstone AM, Flint HJ, Lobley GE. (2007). Reduced dietary intake of carbohydrates by obese subjects results in decreased concentrations of butyrate and butyrate-producing bacteria in feces. Appl Environ Microbiol 73: 1073–1078. 17189447 10.1128/AEM.02340-06 PMC1828662

[bib16] Eaton KA, Honkala A, Auchtung TA, Britton RA. (2011). Probiotic Lactobacillus reuteri ameliorates disease due to enterohemorrhagic Escherichia coli in germfree mice. Infect Immun 79: 185–191. 20974822 10.1128/IAI.00880-10 PMC3019869

[bib17] Faith JJ, Guruge JL, Charbonneau M, Subramanian S, Seedorf H, Goodman AL et al. (2013). The long-term stability of the human gut microbiota. Science 341: 1237439. 23828941 10.1126/science.1237439 PMC3791589

[bib18] Godsall B, Coulson T, Malo AF. (2014). From physiology to space use: energy reserves and androgenization explain home-range size variation in a woodland rodent. J Anim Ecol 83: 126–135. 23931095 10.1111/1365-2656.12116

[bib19] Goodrich JK, Waters JL, Poole AC, Sutter JL, Koren O, Blekhman R et al. (2014). Human genetics shape the gut microbiome. Cell 159: 789–799. 25417156 10.1016/j.cell.2014.09.053 PMC4255478

[bib20] Gurnell J. (1993). Tree seed production and food conditions for rodents in an Oak Wood in southern England. Forestry 66: 291–315.

[bib21] Hayes KS, Bancroft AJ, Goldrick M, Portsmouth C, Roberts IS, Grencis RK. (2010). Exploitation of the intestinal microflora by the parasitic nematode Trichuris muris. Science 328: 1391–1394. 20538949 10.1126/science.1187703 PMC3428897

[bib22] Kabir AM, Aiba Y, Takagi A, Kamiya S, Miwa T, Koga Y. (1997). Prevention of Helicobacter pylori infection by lactobacilli in a gnotobiotic murine model. Gut 41: 49–55. 9274471 10.1136/gut.41.1.49 PMC1027227

[bib23] Keeney KM, Finlay BB. (2011). Enteric pathogen exploitation of the microbiota-generated nutrient environment of the gut. Curr Opin Microbiol 14: 92–98. 21215681 10.1016/j.mib.2010.12.012 PMC3039043

[bib24] Knowles SC, Fenton A, Petchey OL, Jones TR, Barber R, Pedersen AB. (2013). Stability of within-host-parasite communities in a wild mammal system. Proc Biol Sci 280: 20130598. 23677343 10.1098/rspb.2013.0598 PMC3673050

[bib25] Koren O, Goodrich JK, Cullender TC, Spor A, Laitinen K, Backhed HK et al. (2012). Host remodeling of the gut microbiome and metabolic changes during pregnancy. Cell 150: 470–480. 22863002 10.1016/j.cell.2012.07.008 PMC3505857

[bib26] Ley RE, Hamady M, Lozupone C, Turnbaugh PJ, Ramey RR, Bircher JS et al. (2008a). Evolution of mammals and their gut microbes. Science 320: 1647–1651. 18497261 10.1126/science.1155725 PMC2649005

[bib27] Ley RE, Lozupone CA, Hamady M, Knight R, Gordon JI. (2008b). Worlds within worlds: evolution of the vertebrate gut microbiota. Nat Rev Microbiol 6: 776–788. 18794915 10.1038/nrmicro1978 PMC2664199

[bib28] Linnenbrink M, Wang J, Hardouin EA, Kunzel S, Metzler D, Baines JF. (2013). The role of biogeography in shaping diversity of the intestinal microbiota in house mice. Mol Ecol 22: 1904–1916. 23398547 10.1111/mec.12206

[bib29] Maurice CF, Haiser HJ, Turnbaugh PJ. (2013). Xenobiotics shape the physiology and gene expression of the active human gut microbiome. Cell 152: 39–50. 23332745 10.1016/j.cell.2012.10.052 PMC3552296

[bib30] McKnite AM, Perez-Munoz ME, Lu L, Williams EG, Brewer S, Andreux PA et al. (2012). Murine gut microbiota is defined by host genetics and modulates variation of metabolic traits. PLoS One 7: e39191. 22723961 10.1371/journal.pone.0039191 PMC3377628

[bib31] Medellin-Pena MJ, Griffiths MW. (2009). Effect of molecules secreted by Lactobacillus acidophilus strain La-5 on Escherichia coli O157:H7 colonization. Appl Environ Microbiol 75: 1165–1172. 19088323 10.1128/AEM.01651-08 PMC2643578

[bib32] Meyer F, Paarmann D, D'Souza M, Olson R, Glass EM, Kubal M et al. (2008). The metagenomics RAST server - a public resource for the automatic phylogenetic and functional analysis of metagenomes. BMC Bioinformatics 9: 386. 18803844 10.1186/1471-2105-9-386 PMC2563014

[bib33] Moran PA. (1950). Notes on continuous stochastic phenomena. Biometrika 37: 17–23. 15420245

[bib34] Muegge BD, Kuczynski J, Knights D, Clemente JC, Gonzalez A, Fontana L et al. (2011). Diet drives convergence in gut microbiome functions across mammalian phylogeny and within humans. Science 332: 970–974. 21596990 10.1126/science.1198719 PMC3303602

[bib35] Nakagawa S, Schielzeth H. (2013). A general and simple method for obtaining R^2^ from generalized linear mixed-effects models. Methods Ecol Evol 4: 133–142.

[bib36] Nowell F, Higgs S. (1989). Eimeria species infecting wood mice (genus Apodemus) and the transfer of two species to Mus musculus. Parasitology 98: 329–336. 2528109 10.1017/s0031182000061394

[bib37] O'Rourke JL, Grehan M, Lee A. (2001). Non-pylori Helicobacter species in humans. Gut 49: 601–606. 11600455 10.1136/gut.49.5.601 PMC1728516

[bib38] Ochman H, Worobey M, Kuo CH, Ndjango JB, Peeters M, Hahn BH et al. (2010). Evolutionary relationships of wild hominids recapitulated by gut microbial communities. PLoS Biol 8: e1000546. 21103409 10.1371/journal.pbio.1000546 PMC2982803

[bib39] Oksanen J, Blanchet FG, Kindt R, Legendre P, Minchin PR, O'Hara RB et al. (2015), vegan: Community Ecology Package. R package version 2.2-1. http://CRANR-projectorg/package=vegan.

[bib40] Parker SE, Malone S, Bunte RM, Smith AL. (2009). Infectious diseases in wild mice (Mus musculus) collected on and around the University of Pennsylvania (Philadelphia) Campus. Comp Med 59: 424–430. 19887025 PMC2771607

[bib41] Pena JA, Rogers AB, Ge Z, Ng V, Li SY, Fox JG et al. (2005). Probiotic Lactobacillus spp. diminish Helicobacter hepaticus-induced inflammatory bowel disease in interleukin-10-deficient mice. Infect Immun 73: 912–920. 15664933 10.1128/IAI.73.2.912-920.2005 PMC547020

[bib42] Pike N. (2011). Using false discovery rates for multiple comparisons in ecology and evolution. Methods Ecol Evol 2: 278–282.

[bib43] Pritchard MH, Kruse GOW. (1982) The Collection and Preservation of Animal Parasites, vol. 1. University of Nebraska Press: Lincoln, NE.

[bib44] Rausch S, Held J, Fischer A, Heimesaat MM, Kuhl AA, Bereswill S et al. (2013). Small intestinal nematode infection of mice is associated with increased enterobacterial loads alongside the intestinal tract. PLoS One 8: e74026. 24040152 10.1371/journal.pone.0074026 PMC3769368

[bib45] Reynolds LA, Smith KA, Filbey KJ, Harcus Y, Hewitson JP, Redpath SA et al. (2014). Commensal-pathogen interactions in the intestinal tract: lactobacilli promote infection with, and are promoted by, helminth parasites. Gut Microbes 5: 522–532. 25144609 10.4161/gmic.32155 PMC4822684

[bib46] Segata N, Izard J, Waldron L, Gevers D, Miropolsky L, Garrett WS et al. (2012a). Metagenomic biomarker discovery and explanation. Genome Biol 12: R60. 10.1186/gb-2011-12-6-r60 PMC3218848 21702898

[bib47] Segata N, Waldron L, Ballarini A, Narasimhan V, Jousson O, Huttenhower C. (2012b). Metagenomic microbial community profiling using unique clade-specific marker genes. Nat Methods 9: 811–814. 22688413 10.1038/nmeth.2066 PMC3443552

[bib48] Sommer F, Backhed F. (2013). The gut microbiota—masters of host development and physiology. Nat Rev Microbiol 11: 227–238. 23435359 10.1038/nrmicro2974

[bib49] Walk ST, Blum AM, Ewing SA, Weinstock JV, Young VB. (2010). Alteration of the murine gut microbiota during infection with the parasitic helminth Heligmosomoides polygyrus. Inflamm Bowel Dis 16: 1841–1849. 20848461 10.1002/ibd.21299 PMC2959136

[bib50] Wang J, Linnenbrink M, Kunzel S, Fernandes R, Nadeau MJ, Rosenstiel P et al. (2014). Dietary history contributes to enterotype-like clustering and functional metagenomic content in the intestinal microbiome of wild mice. Proc Natl Acad Sci USA 111: E2703–E2710. 24912178 10.1073/pnas.1402342111 PMC4084472

[bib51] Wasimuddin, Cizkova D, Bryja J, Albrechtova J, Hauffe HC, Pialek J. (2012). High prevalence and species diversity of Helicobacter spp. detected in wild house mice. Appl Environ Microbiol 78: 8158–8160. 22961895 10.1128/AEM.01989-12 PMC3485938

[bib52] Watts CHB. (1968). Foods eaten by wood mice (Apodemus sylvaticus) and bank voles (Clethrionomys glareolus) in Wytham Woods, Berkshire. J Anim Ecol 37: 25–41.

[bib53] Wolfe BE, Button JE, Santarelli M, Dutton RJ. (2014). Cheese rind communities provide tractable systems for *in situ* and *in vitro* studies of microbial diversity. Cell 158: 422–433. 25036636 10.1016/j.cell.2014.05.041 PMC4222527

[bib54] Yatsunenko T, Rey FE, Manary MJ, Trehan I, Dominguez-Bello MG, Contreras M et al. (2012). Human gut microbiome viewed across age and geography. Nature 486: 222–227. 22699611 10.1038/nature11053 PMC3376388

[bib55] Zenewicz LA, Yin X, Wang G, Elinav E, Hao L, Zhao L et al. (2013). IL-22 deficiency alters colonic microbiota to be transmissible and colitogenic. J Immunol 190: 5306–5312. 23585682 10.4049/jimmunol.1300016 PMC3646987

