# Supplementary material for: Marked seasonal variation in the wild mouse gut microbiota
Source: ISME J. 2015 May 29;9(11):2423–34. doi: 10.1038/ismej.2015.53 (PMC4611506; doi:10.1038/ismej.2015.53)
Supplement: Supplementary Information [file ismej201553x1.doc]

**Supplementary Information**

**Supplementary Figure Legends**

**Figure S1. Location of woodlands and trapping grids where wood mouse samples were collected.** Both woodlands are situated on the Wirral Peninsula in Cheshire, UK. The location of the six trapping grids used in this study are shown: two in Manor Wood (MW1 and MW2), and four in Haddon Wood (HW1, HW2, HW3 and HW4).

**Figure S2. Relative abundance of bacterial genera in the wild mouse gut microbiota.** Pie charts represent the relative abundance of bacterial genera (n=481 samples). The 21 most abundant genera are shown (the remaining groups are represented by the “other” slice). Taxa are colored based on phylum.

**Figure S3. Seasonal trends in the wild mouse gut microbiota.** Principal coordinates analysis was performed on the Bray-Curtis dissimilarity matrix. The first two coordinates are shown, colored according to season. The indicated *p*-value is from a PERMANOVA analysis (n=112 and 354 samples in the spring and fall, respectively).

**Figure S4. Seasonal patterns are detectable within individuals captured multiple times.** Within-individual changes in Bray-Curtis principle coordinates 1 and 2 (thin lines) track the average population level changes in these metrics (thick lines) in 2010 (panel a) and 2011 (panel b). Thin lines join monthly average scores for individual mice, while thick lines show the population-level average, calculated as the mean value across all mice captured in a particular month.

**Figure S5. Seasonal changes in microbial community structure cannot be explained by changes in overnight temperature.** Black lines represent changes in gut microbial community structure over time, as visualized by Bray-Curtis principle coordinate 1. Grey lines indicate the overnight temperature in degrees celcius over time. Lines were constructed using the geom_smooth function in R package ggplot2 (“loess” method).

**Figure S6. Wild mice and other omnivorous mammals with high levels of Lactobacillales.** Relative abundance of Lactobacillales in captive mammals. Data shown is from a previously published analysis based on 454 pyrosequencing of the V2/V3 region .

**Supplementary Table Legends**

Table S1. 16S rRNA gene sequencing metadata.

Table S2. Metadata used in linear mixed models.

Table S3. Putative bacterial pathogens in the wild mouse gut microbiota.

Table S4. Prevalence of intestinal parasites.

Table S5. Taxonomic groups associated with season.

Table S6. Variance explained by predictor variables in linear mixed models.

**Supplementary Reference**

Muegge BD, Kuczynski J, Knights D, Clemente JC, Gonzalez A, Fontana *L et* al (2011). Diet drives convergence in gut microbiome functions across mammalian phylogeny and within humans*. Scien*c**e 33**2: 970-974.
