# Supplementary figures and images for: Marked seasonal variation in the wild mouse gut microbiota
Source: ISME J. 2015 May 29;9(11):2423–34. doi: 10.1038/ismej.2015.53 (PMC4611506; doi:10.1038/ismej.2015.53)

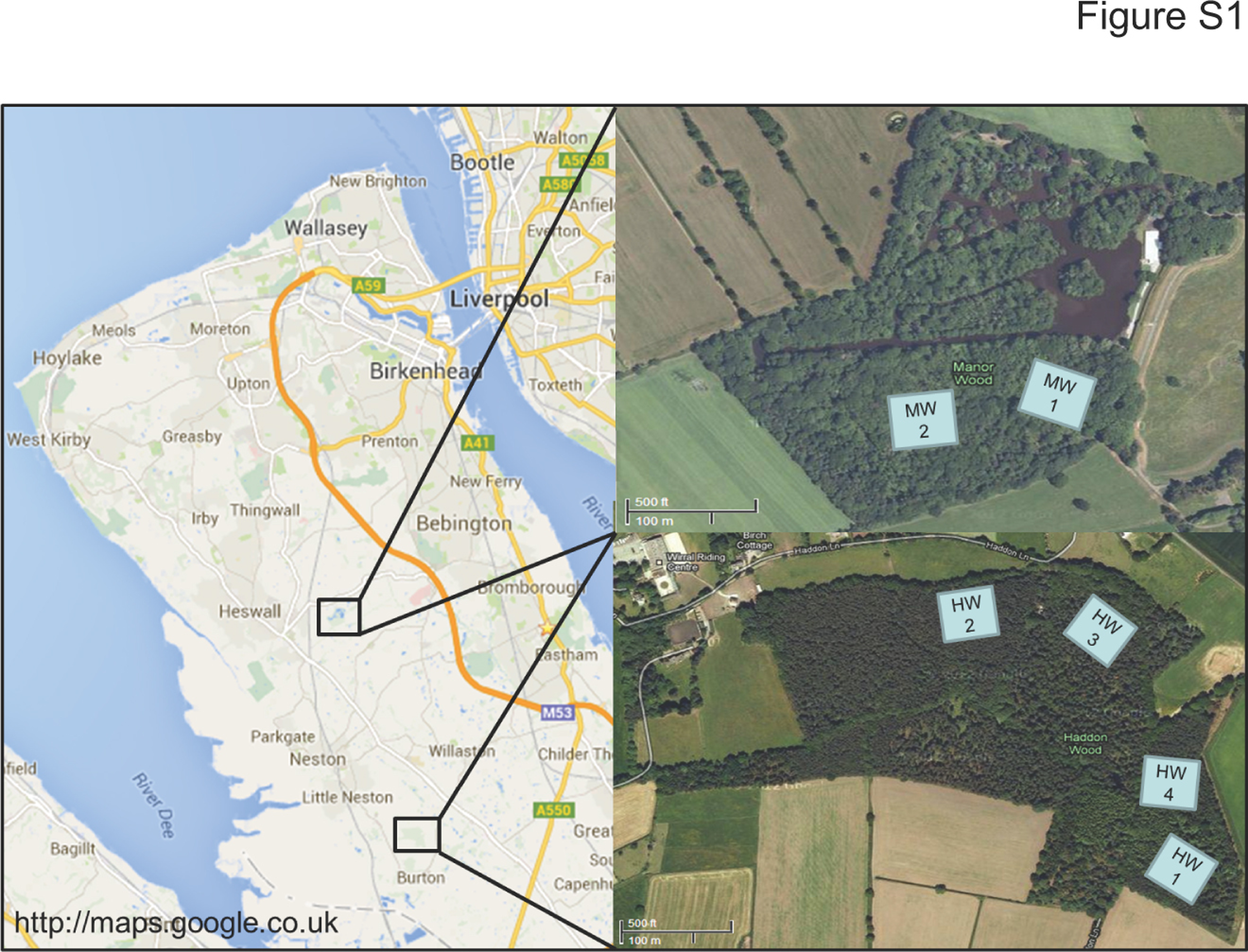

Supplement: Supplementary Figure S1 [file ismej201553x2.tif]

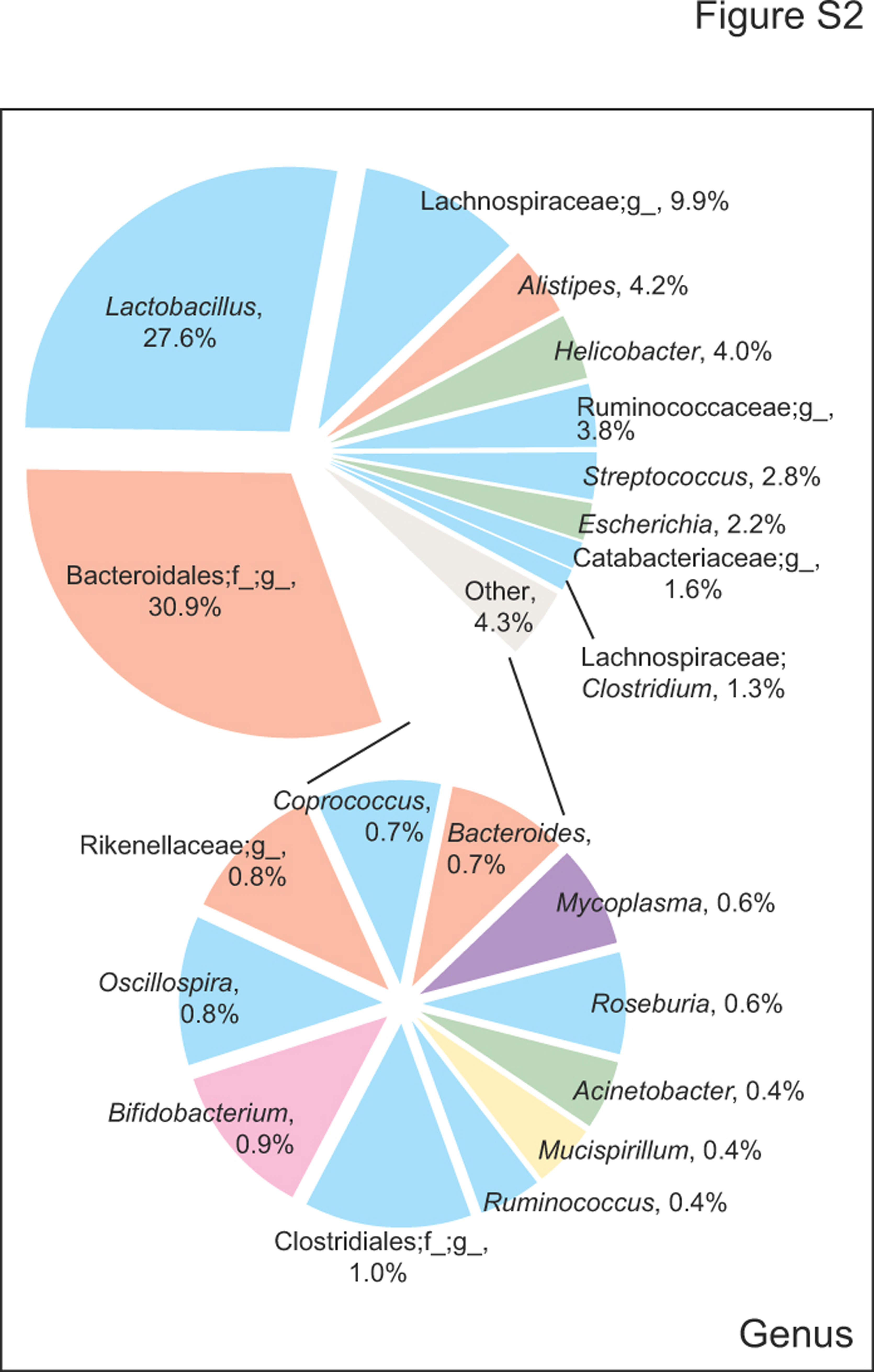

Supplement: Supplementary Figure S2 [file ismej201553x3.tif]

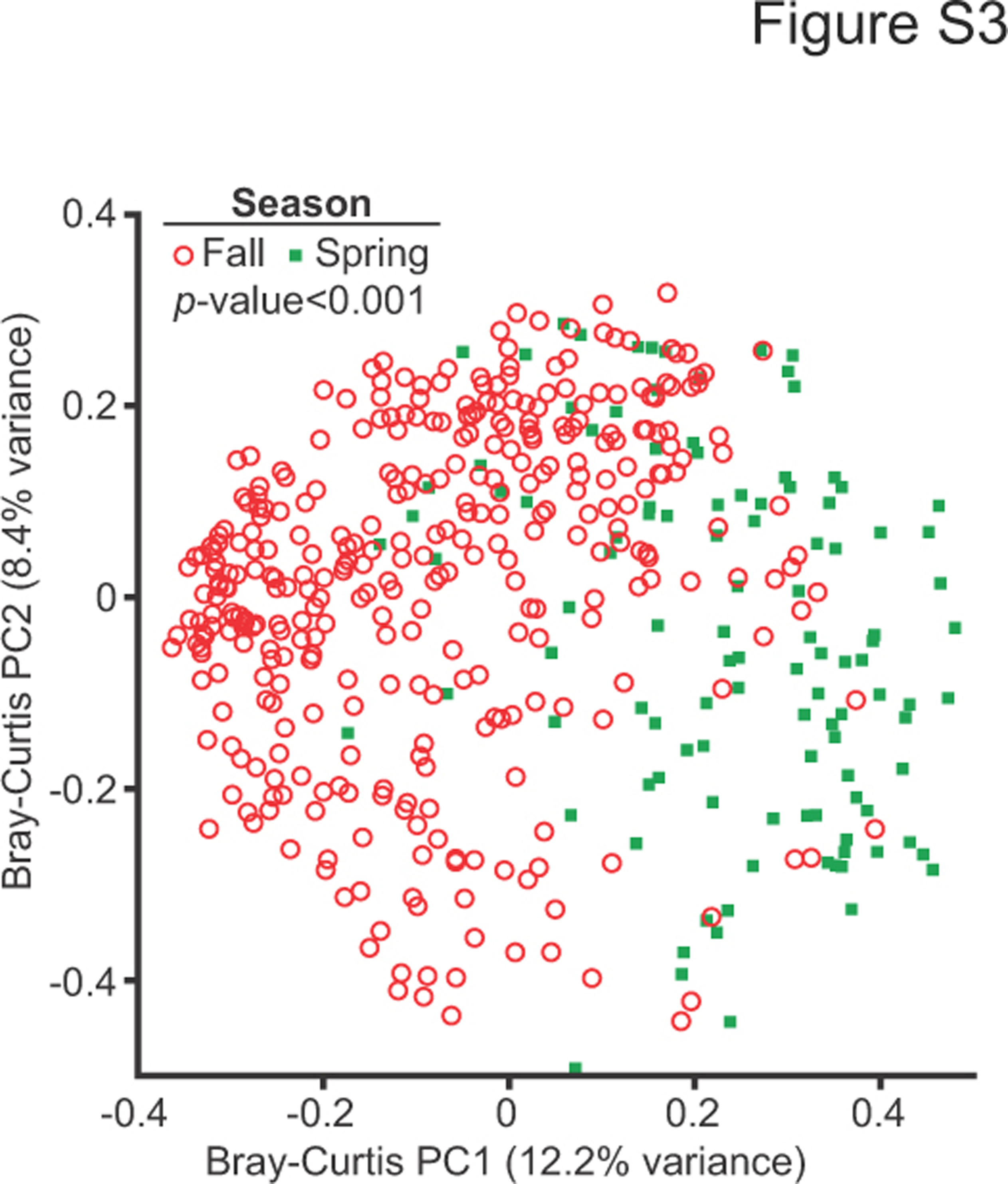

Supplement: Supplementary Figure S3 [file ismej201553x4.tif]

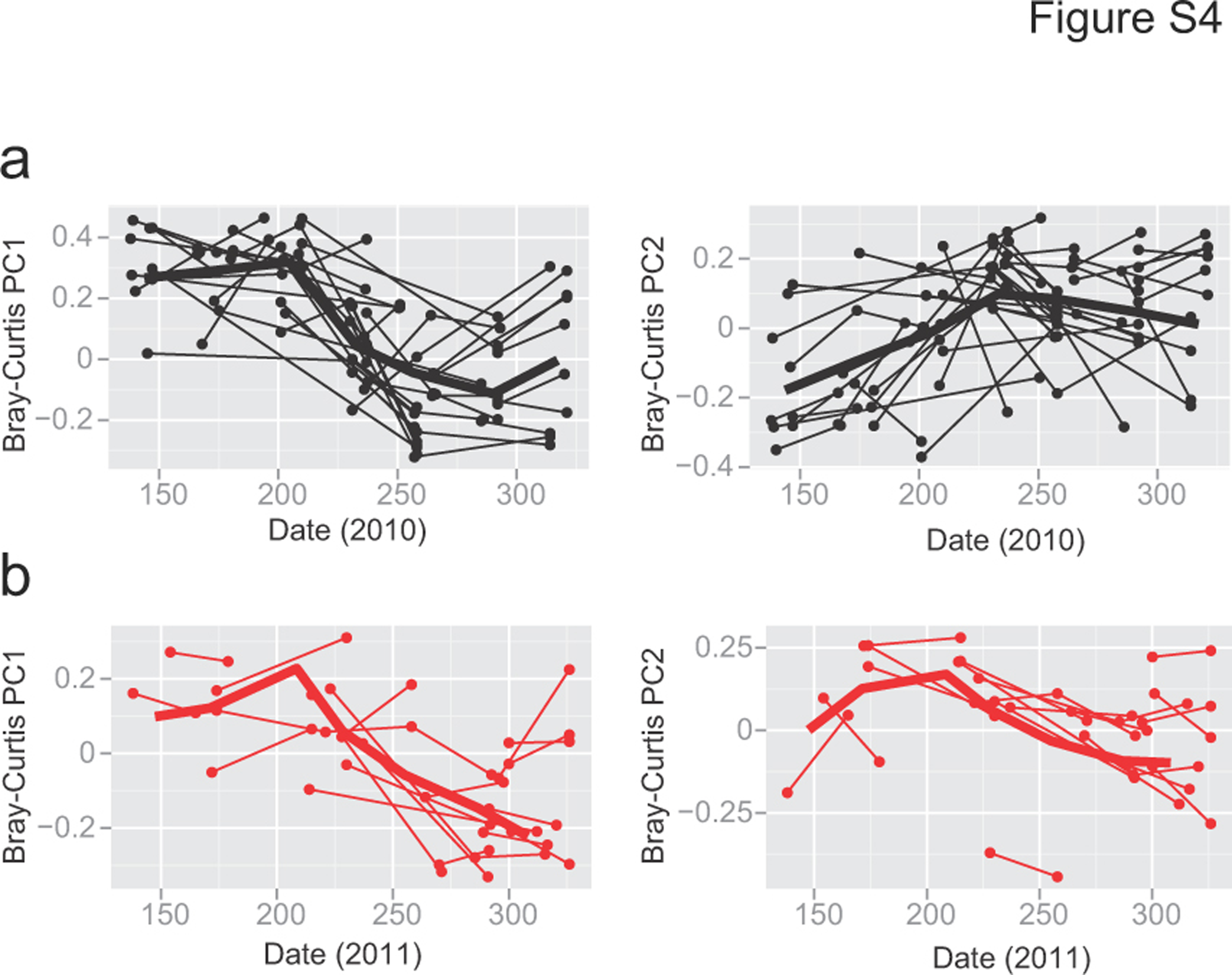

Supplement: Supplementary Figure S4 [file ismej201553x5.tif]

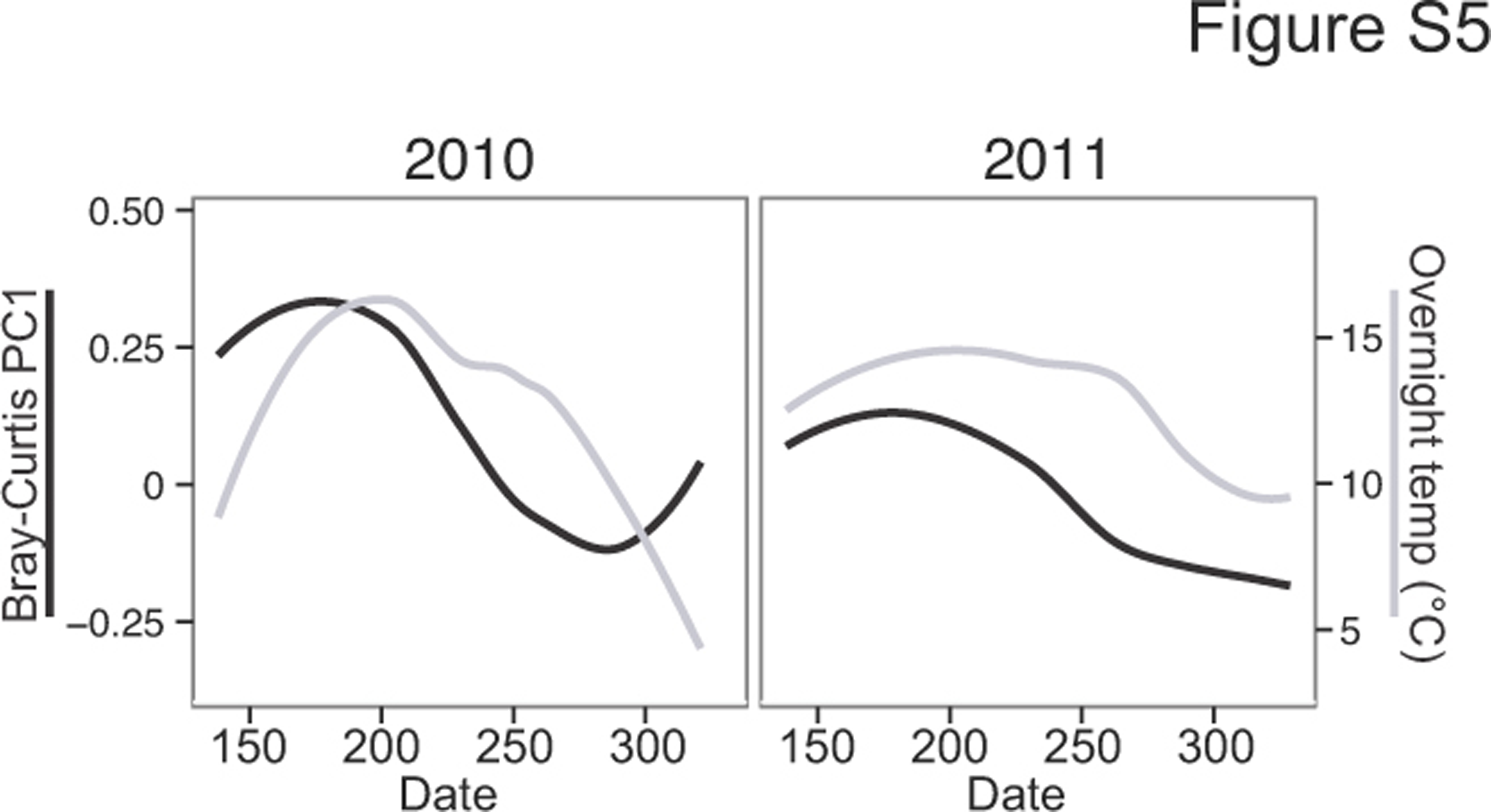

Supplement: Supplementary Figure S5 [file ismej201553x6.tif]

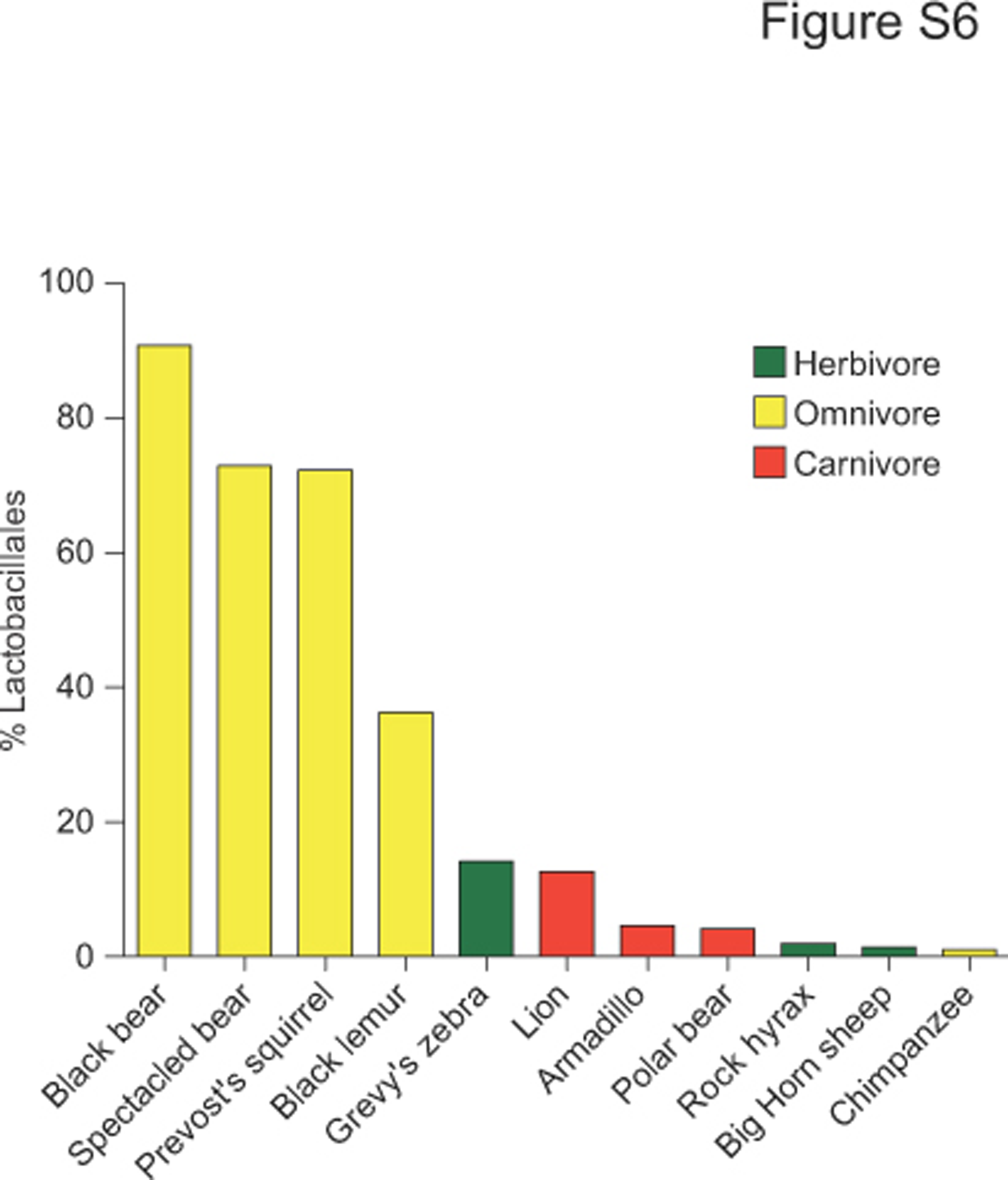

Supplement: Supplementary Figure S6 [file ismej201553x7.tif]
